# Deciphering the Microbial Taxonomy and Functionality of Two Diverse Mangrove Ecosystems and Their Potential Abilities To Produce Bioactive Compounds

**DOI:** 10.1128/mSystems.00851-19

**Published:** 2020-10-27

**Authors:** Shuilin Liao, Yayu Wang, Huan Liu, Guangyi Fan, Sunil Kumar Sahu, Tao Jin, Jianwei Chen, Pengfan Zhang, Lone Gram, Mikael Lenz Strube, Qiong Shi, Simon Ming Yuen Lee, Xin Liu

**Affiliations:** a BGI Education Center, University of Chinese Academy of Sciences, Shenzhen, China; b State Key Laboratory of Agricultural Genomics, BGI-Shenzhen, Shenzhen, China; c BGI-Qingdao, Qingdao, China; d BGI-Shenzhen, Shenzhen, China; e State Key Laboratory of Quality Research in Chinese Medicine, University of Macau, Macao, China; f Institute of Chinese Medical Sciences, University of Macau, Macao, China; g Department of Biotechnology and Biomedicine, Technical University of Denmark, Kongens Lyngby, Denmark; h BGI-Fuyang, Fuyang, China; University of British Columbia

**Keywords:** metagenomics, rhizosphere, biosynthesis gene clusters, antibiotic resistance genes, mangroves

## Abstract

Mangroves, as important and special ecosystems, create unique ecological environments for examining the microbial gene capacity and potential for producing bioactive compounds. However, little is known about the biogeochemical implications of microbiomes in mangrove ecosystems, especially the variations between pristine and anthropogenic mangroves. To elucidate this, we investigated the microbial taxonomic and functional shifts of the mangrove microbiomes and their potential for bioactive compounds in two different coastal mangrove ecosystems in southern China. A gene catalogue, including 87 million unique genes, was constructed, based on deep shotgun metagenomic sequencing. Differentially enriched bacterial and archaeal taxa between pristine mangroves (Guangxi) and anthropogenic mangroves (Shenzhen) were found. The *Nitrospira* and ammonia-oxidizing archaea, specifically, were more abundant in Shenzhen mangroves, while sulfate-reducing bacteria and methanogens were more abundant in Guangxi mangroves. The results of functional analysis were consistent with the taxonomic results, indicating that the Shenzhen mangrove microbiome has a higher abundance of genes involved in nitrogen metabolism while the Guangxi mangrove microbiome has a higher capacity for sulfur metabolism and methanogenesis. Biosynthetic gene clusters were identified in the metagenome data and in hundreds of *de novo* reconstructed nonredundant microbial genomes, respectively. Notably, we found different biosynthetic potential in different taxa, and we identified three high quality and novel *Acidobacteria* genomes with a large number of BGCs. In total, 67,278 unique genes were annotated with antibiotic resistance, indicating the prevalence and persistence in multidrug-resistant genes in the mangrove microbiome.

**IMPORTANCE** This study comprehensively described the taxonomy and functionality of mangrove microbiomes, including their capacity for secondary metabolite biosynthesis and their ability to resist antibiotics. The microbial taxonomic and functional characteristics differed between geographical locations, corresponding to the environmental condition of two diverse mangrove regions. A large number of microbial biosynthetic gene clusters encoding novel bioactivities were found, and this can serve as a valuable resource to guide novel bioactive compound discovery for potential clinical uses.

## Abstract (precis)

This study comprehensively described the taxonomy and functionality of mangrove microbiomes, including their capacity for secondary metabolite biosynthesis and their ability to resist antibiotics. The microbial taxonomic and functional characteristics differed between geographical locations, corresponding to the environmental condition of two diverse mangrove regions. A large number of microbial biosynthetic gene clusters encoding novel bioactivities were found, and this can serve as a valuable resource to guide novel bioactive compound discovery for potential clinical uses.

## INTRODUCTION

Mangrove forests are unique and endangered coastal ecosystems (1), which act as important carbon sinks (“blue carbon”) with global ecological and economic significance. The material, energy, and information flows of the mangroves are dense, showing high productivity, reduction rate, and decomposition rate and significant ecological benefits. The highly productive and diverse microbial community living in the mangrove ecosystem plays an essential role in maintaining the high productivity of mangrove ecosystems and the performance of ecological processes (2).

The structure of the mangrove microbial community is influenced by seasonal changes (3, 4), mangrove species (5), spatial variation, including vertical and horizontal factors (6), and geographical location (7). Environmental variables also contribute to shape the microbial composition, including total nitrogen, total carbon, pH, carbon/nitrogen ratio, and, especially, salinity (8). Zhang et al. (9) have found that mean annual temperature and total organic carbon were the most important factors driving the variations in the mangrove microbiome. In the meantime, anthropogenic and ecological activities (10), especially urbanization and contamination, have huge impact on mangrove sediment microbiome composition (11–13). Due to rapid urbanization, mangroves are often found in or next to urban areas, and anthropogenic activities cause modification of hydrology, sediment, and nutrient dynamics in mangroves (14). These changes can directly disrupt the structure of the microbiota in the mangrove ecosystem. Therefore, it is vital to comprehensively depict sediment microbiome for a better understanding of mangrove functions and resilience.

In southern China, mangroves are found abundantly distributed in Guangxi and Guangdong Provinces. The mangroves in Beilun Estuary National Nature Reserve from Fangchenggang city, Maoweihai Mangrove Nature Reserve from Qinzhou city, and Hepu county from Beihai city in Guangxi Province are located far from industrial or residential areas, and therefore, are considered to be pristine forests (7), mainly comprising various types of true mangroves such as Avicennia marina, Aegiceras corniculatum, Kandelia candel, Bruguiera gymnorrhiza, and Sonneratia apetala. In contrast, Futian National Nature Reserve in Shenzhen of Guangdong Province is the only national nature forest located in an urban area and is mainly represented by the mangroves *Kandelia candel*, *Aegiceras corniculatum*, Acanthus ilicifolius, and *Bruguiera gymnorrhiza*. These two well-separated mangrove ecosystems provide a paradigm to explore the impact of anthropogenic activities on mangrove microbiomes. However, previous studies have been mainly based on amplicon sequencing approaches, which can detect only the taxonomic varieties and not the functional shifts between different ecosystems. To the best of our knowledge, there is no detailed metagenomic research on the microbial communities of Shenzhen mangroves. The unbiased and complete profile of microbial communities mainly covering rhizosphere microbiome in different mangroves in China, or in other regions of the globe, is lacking. Therefore, the purpose of this study was to investigate the taxonomic and functional features of mangrove rhizosphere microbiomes and their shifts between the different locations.

The mangrove ecosystem is becoming a hot spot for studying and discovering novel bioactive secondary metabolites. Over the past 2 decades, more than 1,000 new metabolites have been isolated from mangrove microbes, and of these, ∼850 are derived from endophytic fungi and ∼120 from bacteria (15). Many novel metabolites isolated from mangrove-derived microorganisms have potential for the development of new drugs with antibacterial, antifungal, or antitumor activity (16). However, the diversity and number of secondary metabolites in the mangrove microbiome have not been explored in detail yet.

In this study, we present a robust description of the root-associated microbiome of mangroves located in Guangxi and Shenzhen in China. An 87 million (87M) comprehensive gene catalogue was created based on metagenome data of 39 rhizosphere soil, rhizoplane, and associated bulk soil samples from six representative mangrove plants (see Table S1 in the supplemental material). We hypothesized that geographical locations could explain the majority of variance in the microbial communities of the mangrove ecosystem as anthropogenic activities cause a huge impact on microbial composition (10). We hypothesized that the richness of microbial taxonomy is related to biogeochemical metabolic processes such as the nitrogen, sulfur, and carbon cycles in mangrove sediments, as mangrove sediments were characterized as being carbon rich, sulfur rich, and nitrogen limited (2). We expected to reveal the preferential biogeochemical metabolic processes between the two locations, which represented the changes in ecological functions of mangrove ecosystems under or not under the influence of anthropogenic activities. We also compared the functionalities of the mangrove microbiome with the land and marine ecological environments. We expected to unveil the functional particularity of the mangrove microbiomes as it is the junction between the ocean and the land. Such functionalities must be related with microbes adapted to this special environment. As microbes colonized in a mangrove ecosystem possess significant potential to produce different bioactive secondary metabolites, we expected to identify biosynthetic gene clusters (BGCs) in mangrove microbiomes and characterize the preferred BGC types of different microbial taxa by newly constructed genomes through a bioinformatics approach. This approach provides vital data to facilitate the discovery of bioactive compounds.

10.1128/mSystems.00851-19.4 TABLE S1 Information about rhizosphere, rhizoplane, and bulk soil samples from Guangxi and Shenzhen mangroves. Download Table S1, XLSX file, 0.01 MB. Copyright © 2020 Liao et al. 2020 Liao et al. This content is distributed under the terms of the Creative Commons Attribution 4.0 International license.

## RESULTS

### Microbial gene capacity in mangrove ecosystem.

**FIG 1:**
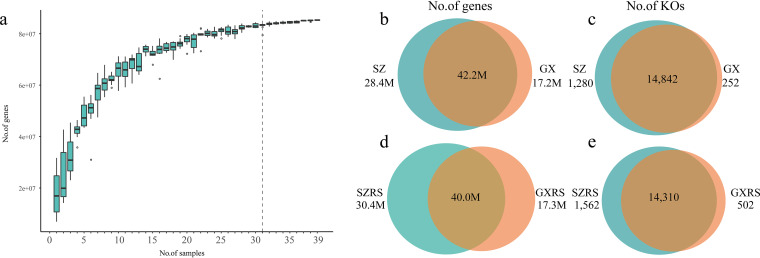
Rarefaction curve of gene numbers and distributions of genes and KOs in two diverse mangrove ecosystems. (a) Rarefaction curve based on the number of detected genes by samplings 10 times. (b and c) The number of shared and unique genes (b) and KOs (c) between 19 Shenzhen samples (SZ) and 20 Guangxi samples (GX). (d and e) The number of shared and unique genes (d) and KOs between 7 SZRS samples and 12 GXRS samples.

We targeted the rhizospheric microbiome samples from the coastal mangrove ecosystem situated along the coasts of the southern China provinces of Guangdong and Guangxi. We collected 19 rhizosphere, rhizoplane, and associated bulk soil samples from four representative mangrove plants in the Shenzhen Mangrove National Nature Reserve in Guangdong Province. We collected a further 20 rhizosphere and associated bulk soil samples from five representative mangrove plants from three mangrove wetlands of the Beilun Estuary, Hepu and Maoweihai in Guangxi Province (Table 1; see also Table S1 in the supplemental material). The study generated 1,701 gigabases (Gb) of metagenomic data with an average of 43.6 Gb data per sample. On average, 25.9% of the reads were utilized for metagenomic contig construction, which generated 57.37 Gb assembled metagenome sequences. Approximately 186 million (186M) genes were predicted from metagenomic sequences and then were clustered into a nonredundant gene set, comprising 87.8M unique genes, here referred to as the mangrove root microbial gene catalogue. A range of 16.79% to 51.75% reads from each sample were mapped to this catalogue, enabling the detection of 14.74M to 45.43M unique genes in individual samples (Table S2). From the rarefaction curves for gene numbers, we observed that the number of detected genes began to reach the saturation stage from 31 or more in our deeply sequenced samples (Fig. 1a). Similar to the above observation, the rarefaction curves of gene numbers from pristine mangroves (Guangxi) (Fig. S1a) and anthropogenic mangroves (Shenzhen) (Fig. S1b) both reached toward the plateau, suggesting that the sampling effort covered most of the microbial genes in each mangrove ecosystem. A total of 42.2M microbial genes were shared between the Shenzhen mangrove and Guangxi mangrove ecosystem, constituting approximately half of the genes of the catalogue. However, 28.4M genes were unique in Shenzhen, while 17.2M genes were unique in Guangxi (Fig. 1b). Meanwhile, 14,842 KEGG Orthologs (KOs), 90.64% of the totals (16,373 KOs), were shared by both locations, with 1,280 being unique in Shenzhen and 252 unique in Guangxi (Fig. 1c). A large number of unique microbial genes and KOs were identified in the Shenzhen mangrove ecosystem, and similar variations were found in mangrove rhizosphere samples between Shenzhen and Guangxi (Fig. 1d and e). However, the α-diversity of rhizosphere samples in Shenzhen was significantly higher than that in Guangxi (Shannon index based on functional genes, Fig. S1c, *P* = 0.034).

**TABLE 1:** Detailed information of mangrove habitats where sampling was conducted in this study

Group	Sample site	Geographical location	Longitude and latitude	Natural condition	Plant species^*a*^	Compartments
Guangxi	GB	Hepu county of Beihai city	21°36'12.3′N 108°59'37.8"E	Far from city	AC, AM, KC, SA	Rhizosphere, bulk soil
	GE	Beilun Estuary National Nature Reserve	21°36'54.7"N 108°13'56.3′E	Far from city	AC, AM, BG, KC	Rhizosphere, bulk soil
	GM	Maoweihai Mangrove Nature Reserve	21°52'00.0"N 108°34'55.9"E	Far from city	AC, KC	Rhizosphere, bulk soil
Shenzhen	SU	Futian Mangrove Nature Reserve (constructed wetland system)	22°31'41.1"N 113°59'55.3′E	Constructed wetland system for treating wastewater	AC, KC	Rhizosphere, rhizoplane
	S	Futian Mangrove Nature Reserve	22°31'41.1"N 113°59'55.3′E	In the hinterland of the city	AC, AI, BG, KC	Rhizosphere, rhizoplane, bulk soil

a Plant species abbreviations: AC, *Aegiceras corniculatum*; AM, *Avicennia marina*; KC, *Kenaelia candel*; SA, *Sonneratia apetala*; BG, *Bruguiera gymnorrhiza*; AI, *Acanthus ilicifolius*.

10.1128/mSystems.00851-19.1 FIG S1 Rarefaction curves of gene numbers and alpha diversity in two diverse mangrove ecosystems. (a) Rarefaction curves of gene numbers from pristine mangroves (Guangxi). (b) Rarefaction curves of gene numbers from anthropogenic mangroves (Shenzhen). (c) Comparison of alpha diversity between the bulk soil and rhizosphere samples from each location based on the Shannon index. Download FIG S1, PDF file, 0.2 MB. Copyright © 2020 Liao et al. 2020 Liao et al. This content is distributed under the terms of the Creative Commons Attribution 4.0 International license.

10.1128/mSystems.00851-19.5 TABLE S2 Read mapping information of each sample in the mangrove root microbial gene catalogue. Download Table S2, XLSX file, 0.01 MB. Copyright © 2020 Liao et al. 2020 Liao et al. This content is distributed under the terms of the Creative Commons Attribution 4.0 International license.

### Taxonomic features of the mangrove rhizosphere microbiome.

**FIG 2:**
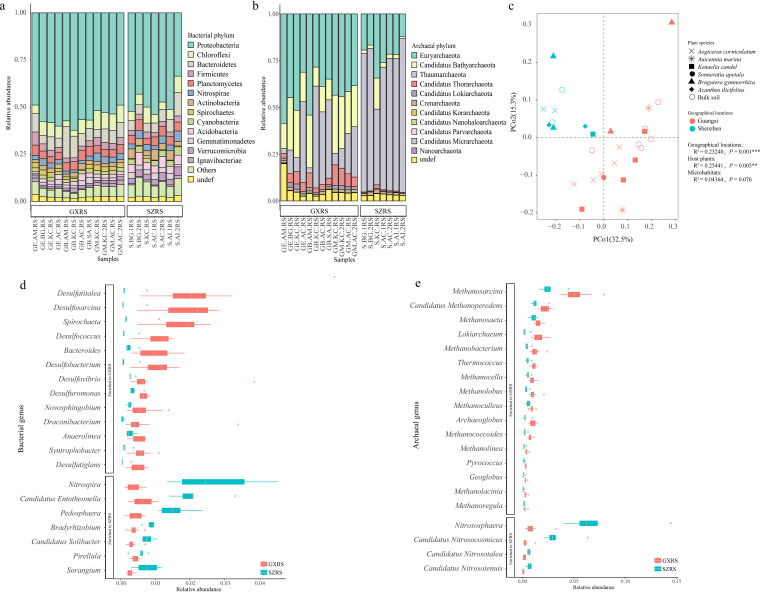
The taxonomic composition of the mangroves’ rhizosphere microbiome varied between the different locations. (a and b) Rhizosphere microbial community composition at the phylum level. Only the microbial phyla with a relative abundance in the top 10% of bacteria (a) and all archaeal phyla (b) were shown. (c) PCoA analysis based on genus abundance profile was performed to assess the influences of geographical location, microhabitat, and host plant on mangrove root microbial communities. (d and e) Differential enrichment of bacterial and archaeal genus between different locations were identified using a Wilcoxon rank sum test with an adjusted *P* value of <0.05. Only the top 20 enriched bacterial genera (d) and the top 20 enriched archaeal genera (e) are shown in the figure.

A large fraction (67.01%) of genes in the mangrove microbiome originated from bacteria, while only 2.01%, 0.2%, and 0.16% were annotated to archaea, eukaryotes, and viruses, respectively. The remaining 30.62% were unknown genes, which could be derived from novel or unknown taxa. Bacteria were the most abundant domain recovered from all rhizosphere samples, ranging from 47.67% to 82.28% of the total sequence abundance, followed by archaea ranging from 1.39% to 5.71% and eukaryotes ranging from 0.09% to 1.87%, respectively. A total of 83 bacterial phyla (Table S3), 1,571 bacterial genera, 11 archaeal phyla (Table S3), 118 archaeal genera, 9 fungal phyla, and 220 fungal genera were detected in all the rhizosphere samples. Among all the bacterial phyla in the rhizosphere samples, *Proteobacteria* accounted for 33.55% to 62.20% of the sequence abundance, followed by *Bacteroidetes* (4.12% to 16.54%), *Chloroflexi* (3.15% to 10.23%), *Planctomycetes* (3.29% to 5.63%), and *Actinobacteria* (1.42% to 5.63%) (Fig. 2a, Table S3). Although *Proteobacteria* was the predominant phylum in all rhizosphere soil samples, the class compositions varied in both the studied locations. *Proteobacteria* was mainly comprised of *Alphaproteobacteria*, *Gammaproteobacteria*, *Betaproteobacteria*, and Deltaproteobacteria, which contributed to an average of 27.86%, 27.11%, 22.38%, and 21.05% in Shenzhen mangrove rhizosphere samples (SZRS), respectively. However, *Proteobacteria* in Guangxi mangrove rhizosphere samples (GXRS) was mainly represented by Deltaproteobacteria, accounting for an average 43.85% of the total sequences (Fig. S2a). Meanwhile, *Thaumarchaeota* was the most abundant archaeal phylum in SZRS, whereas *Euryarchaeota* was the most abundant archaeal phylum in GXRS (Fig. 2b, Table S3). In fungal composition, Ascomycota was the most abundant fungal phylum, followed by Basidiomycota, Glomeromycota, Chytridiomycota, and Microsporidia (Fig. S2b).

10.1128/mSystems.00851-19.2 FIG S2 Taxonomic composition of the mangroves’ rhizosphere microbiome at the class level of *Proteobacteria* and fungal phylum level. (a) Taxonomic composition of the rhizosphere microbiome in Guangxi and Shenzhen mangroves at the class level of *Proteobacteria* phylum. (b) Rhizosphere microbial community composition at the fungal phylum level. Download FIG S2, PDF file, 0.2 MB. Copyright © 2020 Liao et al. 2020 Liao et al. This content is distributed under the terms of the Creative Commons Attribution 4.0 International license.

10.1128/mSystems.00851-19.6 TABLE S3 Relative abundance of all 83 bacterial phyla and 11 archaeal phyla in 19 rhizosphere samples from Guangxi and Shenzhen mangroves. Download Table S3, XLSX file, 0.03 MB. Copyright © 2020 Liao et al. 2020 Liao et al. This content is distributed under the terms of the Creative Commons Attribution 4.0 International license.

The correlations between geographical locations (the Shenzhen and Guangxi mangroves), microhabitats (the habitats of the microorganism derived from bulk soil or rhizosphere), and host plants (the mangrove trees, from where the rhizosphere samples were collected) with the bacterial community of the mangrove ecosystem were explored. Unconstrained principal coordinate analysis (PCoA) was performed in this exploration to uncover the separate patterns between microbial communities based on the Bray-Curtis distance of taxonomic genus abundance. The majority of variations in microbial composition were explained by geographical locations and host plants (Fig. 2c). Permutational multivariate analysis of variance (PERMANOVA) also corroborated that geographical locations and host plants comprised 48.6% of the variation within the microbiome data (23.2%, *P* = 0.001; 25.4%, *P* = 0.008, respectively). However, no significant difference in microbial communities between microhabitats was observed.

Interestingly, 540 bacterial genus abundances such as *Nitrospira*, “*Candidatus* Entotheonella”, *Pedosphaera*, *Bradyrhizobium*, and “*Candidatus* Solibacter” were statistically higher in SZRS than in the GXRS, while 229 bacterial genus abundances such as *Desulfatitalea*, *Desulfosarcina*, *Spirochaeta*, *Desulfococcus*, and *Desulfobacterium* were observed in GXRS (Fig. 2d, Table S4). Among the 229 genera, 32 genera with a total relative abundance ranged from 3.61% to 18.85% in GXRS, belonged to sulfate-reducing bacteria (SRB). Moreover, seven archaeal genus abundances were statistically higher in the SZRS than in the GXRS. Among the seven SZRS enriched genera, five genera (*Nitrososphaera*, “*Candidatus* Nitrosocosmicus,” “*Candidatus* Nitrosotalea,” “*Candidatus* Nitrosotenuis,” and “*Candidatus* Nitrosopelagicus”) were ammonia-oxidizing archaea (AOA), which may play a crucial role in soil nitrogen cycling (Fig. 2e, Table S4). On the other hand, among the 47 GXRS enriched archaeal genera, 32 genera such as *Methanosarcina*, “*Candidatus* Methanoperedens,” *Methanosaeta*, and *Methanobacterium*, with a total relative abundance of from 12.99% to 29.91% in GXRS, belonged to the methanogens (Fig. 2e, Table S4). In addition, three fungal genera such as *Pyronema*, *Trichophyton*, and *Suillus* were found to be enriched in the SZRS.

10.1128/mSystems.00851-19.7 TABLE S4 Relative abundance of genera enriched in Guangxi rhizosphere samples and Shenzhen rhizosphere samples. Bacterial genera shown on yellow background indicated SRB. Archaeal genera shown on yellow background indicated methanogenic archaea, and archaeal genera on green background indicated AOA. Download Table S4, XLSX file, 0.2 MB. Copyright © 2020 Liao et al. 2020 Liao et al. This content is distributed under the terms of the Creative Commons Attribution 4.0 International license.

### Functional trait variations between GXRS and SZRS microbiome.

**FIG 3:**
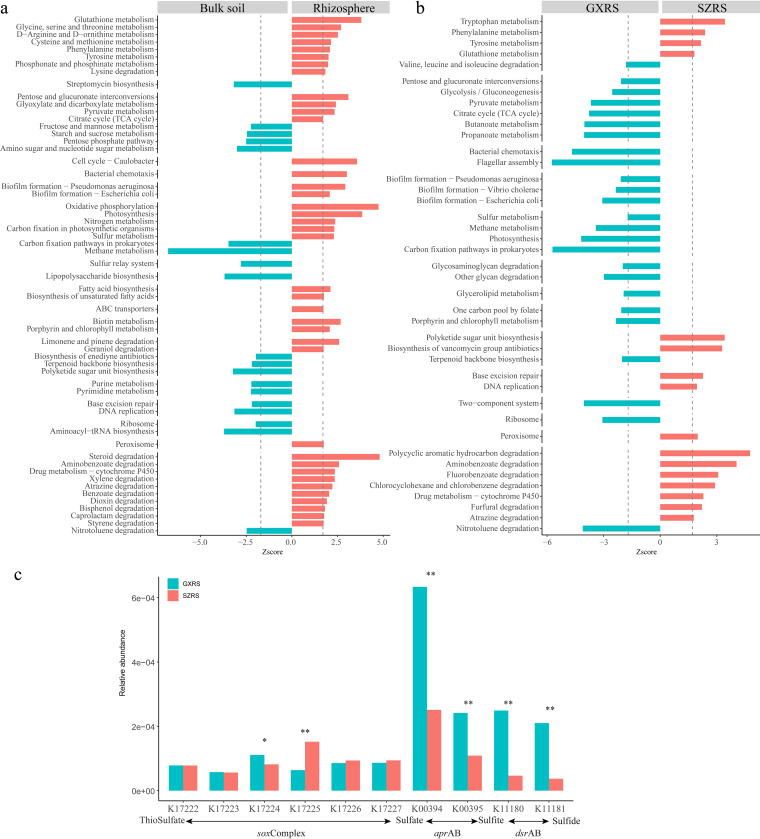
KEGG pathways enriched in mangrove rhizosphere soil samples. (a) KEGG pathways compared between mangrove rhizosphere samples and correspondent bulk soil samples. (b) KEGG pathways compared between SZRS and GXRS. Pathways with a significant difference in z-score ≥ 1.7 or z-score < −1.7 are shown. (c) Sulfur metabolism profile in sulfur oxidation and sulfate reduction. Statistical differences between the two mangrove groups evaluated by Wilcoxon rank sum test are indicated by asterisks as follows: *, *P* value of <0.05; **, *P* value of <0.01. Gene abbreviations: *sox*Complex, sulfur-oxidizing protein complex; *apr*AB, adenylylsulfate reductase, subunit A/B; *dsr*AB, dissimilatory sulfite reductase alpha/beta subunit.

Pairwise comparison of rhizosphere samples with corresponding bulk soil samples revealed enriched KOs in the rhizosphere microbiome. These enriched KOs mainly contributed to 38 KEGG-specific pathways, which were significantly enriched in the rhizosphere microbiome. These enrichments were evident from the reporter scores (Fig. 3a; adjusted *P* value < 0.05, z-score ≥ 1.7). We defined these pathways as the core functional traits of the mangrove rhizosphere microbiome. Of these functional traits, oxidative phosphorylation, photosynthesis, nitrogen metabolism, and sulfur metabolism were significantly elevated in the rhizosphere microbiome, and these contributed to the organic compound synthesis, energy metabolism, and nutrition cycling. Bacterial chemotaxis and biofilm formation involved in environmental adaptation and microbe-microbe interactions were overrepresented in the rhizosphere microbiome compared to the bulk soil microbiome. In addition, the ATP-binding cassette (ABC) transporter system, which mainly contributes to nutrient exchange, was also present in higher levels in the rhizosphere microbiome. A total of nine subpathways in xenobiotic biodegradation and catabolism pathways were found to be involved in the degradation of aromatic compounds such as aminobenzoate, steroid, citalopram, xylene, atrazine, benzoate, dioxin, bisphenol, caprolactam, and styrene in the rhizosphere microbiome. These aromatic compounds may be released by plants to defend against plant pathogens. In addition, we detected two subpathways of terpenoid and polyketide metabolism, involved in degrading compounds with antibacterial activity such as limonene, pinene, and geraniol, which may come from mangrove root exudates (18). Many of the core rhizosphere functions are related to the nutritional acquisition, environmental adaptation, and pathogenic inhibition to protect plants, and some of these functions have also been reported for the citrus and barley root microbiome (19, 20).

The functional differences of the rhizosphere microbiome in Shenzhen and Guangxi mangroves were also evaluated using functional comparison to describe the impact of anthropogenic activities. Sixteen functional pathways were enriched in SZRS, while 25 pathways were enriched in GXRS (Fig. 3b; adjusted *P* value < 0.05, |z-score| ≥ 1.7). The functional pathways were related to amino acid metabolism and DNA replication and repair. Metabolism of terpenoids and polyketides and biodegradation and metabolism of xenobiotics were mainly enriched in SZRS. However, the pathways involved in energy metabolism (such as carbon fixation pathways, photosynthesis, methane metabolism, and sulfur metabolism), carbohydrate metabolism, cell motility (flagellar assembly and bacterial chemotaxis), and translation and signal transduction (two-component system) were mainly enriched in the GXRS. The differentially enriched pathways, especially carbon metabolism, sulfur metabolism, and xenobiotic biodegradation and metabolism pathways between two locations, indicated the change in ecological function of the mangrove microbiome.

The microbial biogeochemical transformations of methane, nitrogen, and sulfur in two mangrove ecosystems were specifically analyzed (Fig. S3). In three different reactions of methanogenesis (CO_2_ to methane, methanol to methane, and acetate to methane), the *hdr* gene involved in the formation of methane showed the highest abundance in GXRS (Fig. S3a). Compared to methanogenesis, the average abundances of the genes involved in methane oxidation were much lower. In nitrogen metabolism, genes related to the dissimilatory nitrate reduction (DNRA), assimilatory nitrate reduction (ANRA), denitrification, nitrification, nitrogen fixation, and anammox were observed (Fig. S3b). Of these genes, genes encoding enzymes such as *nar*, *nir*, *nor*, *nxr*, and *amo* were enriched in SZRS (Fig. S3b). In sulfur metabolism (Fig. S3c), genes related to sulfate reduction processes predominated over oxidization reactions. The abundances of genes involved in the reaction of sulfate into APS to sulfite were more abundant than those involved in the 3′-phosphoadenosine-5′-phosphosulfate (PAPS)-to-sulfite pathways. Moreover, the sulfate reduction genes *apr*AB (adenylylsulfate reductase, subunit A/B) (K00394 and K00395) and *dsr*AB (dissimilatory sulfite reductase alpha/beta subunit) (K11180 and K11181) were significantly higher in GXRS than in SZRS (Fig. 3c).

10.1128/mSystems.00851-19.3 FIG S3 Biochemical transformations of methane, nitrogen, and sulfur in mangrove microbiome. (a) Methane metabolism. (b) Nitrogen metabolism. (c) Sulfur metabolism. Boxes indicate the related genes involving the transformation, and the genes with an asterisk represent a series of genes. Numbers in circles indicate the sum of the absolute abundance of genes in different locations. (d) The number of genes involved in different KEGG functional categories. Gene abbreviations: *hdr*, heterodisulfide reductase; *nar*, nitrate reductase; *nir*, nitrite reductase; *nor*, nitric oxide reductase; *nxr*, nitrite oxidoreductase; *amo*, ammonia monooxygenase. Download FIG S3, PDF file, 1.0 MB. Copyright © 2020 Liao et al. 2020 Liao et al. This content is distributed under the terms of the Creative Commons Attribution 4.0 International license.

### Microbial functional particularity of the mangrove ecosystem compared to land and ocean ecosystems.

**FIG 4:**
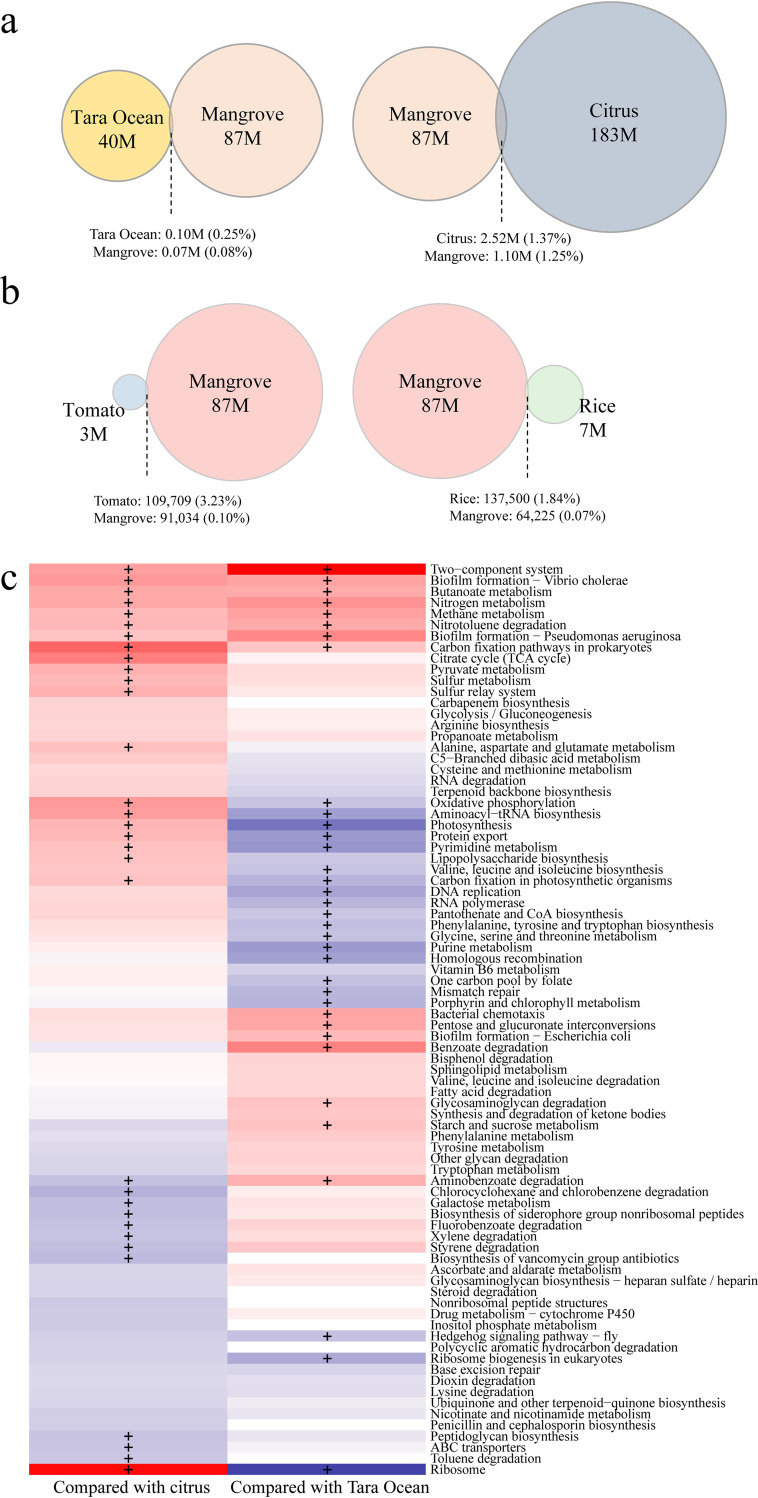
Comparisons of microbial gene compositions and KEGG pathways between mangrove and other ecosystems. (a) Mangrove microbial gene set compared with that from Tara Ocean microbiome and citrus root microbiome. M, million. (b) Mangrove microbial gene set compared with that from model plant rice and tomato root microbiome. (c) KEGG pathways enriched in mangrove rhizosphere samples compared with citrus rhizosphere soil samples (left column) and Tara Ocean surface samples (right column), respectively. Only pathways with a significant difference in reporter scores were retained. Pathways with z-score < −1.7 and z-score > 1.7 were colored blue and red, meaning pathways enriched in other ecosystems and mangrove rhizosphere soil, respectively. A + symbol indicates a |z-score| of >2.58.

Mangrove forests usually grow near the fringe of land and ocean and are characterized by the regular inundation of tidal water. They are a special ecosystem of land-to-sea transition. In order to characterize the microbial functions of this ecosystem, we compared the microbial gene content and functional composition of mangrove forest with land soil and sea samples. One is the global citrus root-associated microbiome unique gene set (19), representing the root-associated microbiome of a kind of terrestrial plant, while the other is the Tara Ocean gene set (21), representing the microbiome of the marine ecosystem. Only 71,162 (0.08%) genes from our gene set were mapped to the Tara Ocean, accounting for an average of 0.16% of gene abundance in mangrove samples. However, 1.10M (1.25%) genes from the mangrove gene set were mapped to the citrus gene set (Fig. 4a), accounting for an average of 1.70% of gene abundance. A greater number of genes were overlapping between mangrove data and citrus data than in the case of the Tara Ocean. We also compared the mangrove gene set with limited metagenome root microbiome data from model plant rice (22) and tomato (23), and only 64,225 (0.07%) and 91,034 (0.1%) genes from the mangrove microbial gene set were mapped to the rice and tomato microbial gene set, respectively (Fig. 4b). These results indicated that there are fewer common genes between mangroves and non-woody plants, which further validated the uniqueness of the mangrove ecosystem

The comparative functional analysis from 19 rhizosphere samples of mangrove forests with 20 rhizosphere samples of citrus and 63 surface samples of the Tara Ocean showed that 32 and 25 functional pathways were enriched in mangrove rhizosphere samples compared with land soil and sea, respectively (Fig. 4c, adjusted *P* value < 0.05, |z-score| > 1.7). Of these functional pathways, eight pathways were commonly enriched in the mangrove ecosystem compared with land and sea. These pathways mainly comprised carbon fixation pathways, butanoate metabolism, nitrogen metabolism, methane metabolism, two-component systems, biofilm formation, and nitrotoluene degradation in prokaryotes (adjusted *P* value < 0.05, |z-score| > 2.58). These commonly enriched pathways are mainly involved in biochemical cycling, such as carbon, nitrogen, and methane metabolism and microbe-microbe interactions. These interactions may contribute to the special environment of mangrove forests with their anaerobic ecosystems, rich in organic matter. Such an ecosystem is optimal for anaerobic microorganisms, such as AOA and methanogenic archaea, which are responsible for nutrient cycling.

### Microbial bioactive secondary metabolites in the mangrove ecosystem.

**FIG 5:**
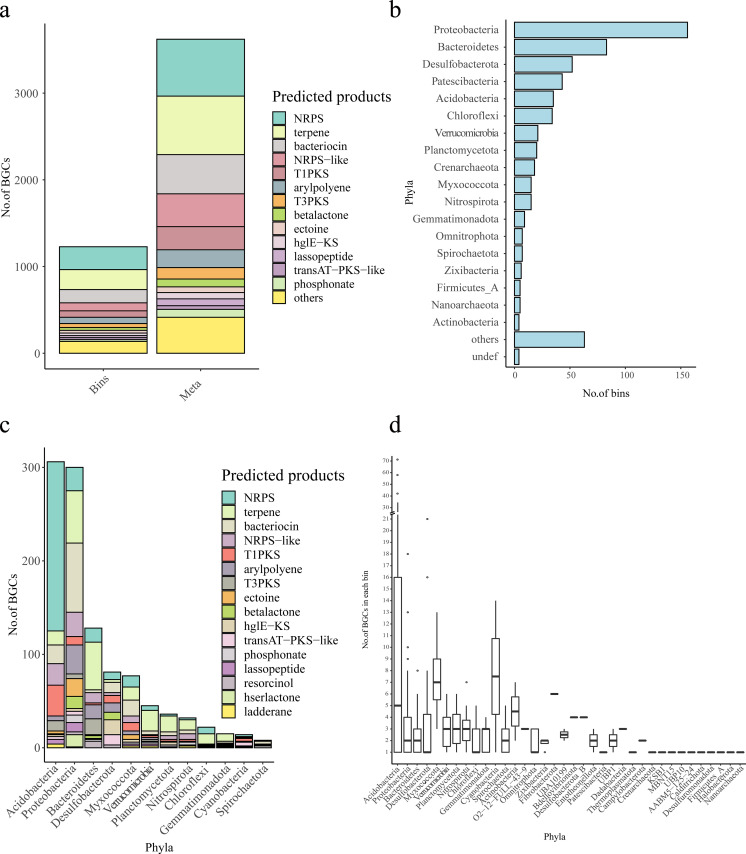
Information on secondary metabolite BGCs in metagenome data and bins. (a) BGC composition in metagenome data (Meta) and in 602 newly reconstructed bins (Bins). (b) Taxonomic information of 602 bins at the phylum level. (c) Distribution of different BGCs in phyla. (d) BGC numbers per genome in different phyla.

In the metagenome data, a total of 3,622 secondary metabolite BGCs were identified using antiSMASH5.0. In total, 675, 656, 452, 379, 267, 205, 132, 90, and 766 BGCs were inferred to synthesize terpene, nonribosomal peptide synthetase (NRPS), bacteriocins, NRPS-like, type I polyketide synthases (T1PKS), arylpolyene, type III polyketide synthases (T3PKS), betalactone, and other products, respectively (Fig. 5a). Of these, terpene and NRPS were the two largest classes of BGCs from the mangrove ecosystem. In 3,622 BGCs, only 761 gene clusters (21.01%) were known and had homolog gene clusters in the MIBiG, encoding 174 different bioactive compounds (Table S5). Most of these compounds, such as carotenoid (24), flexirubin (25), ectoine (26), and rhizomide (27), have been reported to have pharmacological functions, such as antioxidants, antimicrobial, enzyme stabilizer, or anticancer activities. Metagenome data provide us with a foundation to mine biosynthesis clusters at the contig level.

10.1128/mSystems.00851-19.8 TABLE S5 Detailed information of 761 known BGCs in metagenome data and 256 known BGCs in newly reconstructed bins. Download Table S5, XLSX file, 0.01 MB. Copyright © 2020 Liao et al. 2020 Liao et al. This content is distributed under the terms of the Creative Commons Attribution 4.0 International license.

To further evaluate the potential for producing bioactive compounds in different microbial taxa from the mangrove ecosystem, we targeted the newly reconstructed 602 nonredundant assembled genomes from our metagenome data, which covered 42 bacterial phyla (567 bins) and six archaeal phyla (31 bins). Phylogenetic annotations showed that 165 bins were affiliated with *Proteobacteria*, and 83 were from *Bacteroidetes*, 52 from *Desulfobacterota*, 43 from *Patescibacteria*, 35 from *Acidobacteria*, 34 from *Chloroflexi*, and 21 from *Verrucomicrobia* (Fig. 5b, Table S6). In total, 1,228 BGCs were predicted from 36 phyla covered by 328 bins. The most common and abundant classes of BGCs identified in these genomes were consistent with metagenome data, including NRPS, terpene, and bacteriocin biosynthesis. However, the classes of BGCs found in the genomes varied across the taxonomic group. Some phyla had higher proportions of certain BGC classes (Fig. 5c). For example, NRPS (181 BGCs) and T1PKS (33 BGCs) dominated in the *Acidobacteria* genomes, while bacteriocin (74 BGCs) and terpene (56 BGCs) were well represented in the genomes of *Proteobacteria*. Terpene (51 BGCs) was also abundant in *Bacteroidetes* genomes. hglE-KS (16 BGCs) was mainly found in *Desulfobacterota*. Interestingly, we found that *Acidobacteria* was the taxon with the most abundant gene clusters, followed by *Proteobacteria*, *Bacteroidetes*, and *Desulfobacterota* and *Myxococcota*, though the genome numbers and abundance of *Acidobacteria* were lower than those of *Proteobacteria* and *Bacteroidetes*. We also found that *Acidobacteria*, *Myxococcota*, *Cyanobacteria*, and *Actinobacteria* exhibited a higher median value of BGCs per genome than other phyla (Fig. 5d). In particular, three nearly complete genomes, Acidobacteria_bin4, Acidobacteria_bin9, and Acidobacteria_bin15, in *Acidobacteria* were identified with exceptionally high numbers of BGCs, 42, 71, and 58, respectively (Table 2). Acidobacteria_bin9 and Acidobacteria_bin15 were from the class *Aminicenantia*, and Acidobacteria_bin4 was from GCA-2747255. As expected, NRPS and PKS were the two largest classes of BGCs in the three genomes, but a smaller number of other class BGCs such as bacteriocin, betalactone, thiopeptide, and so on, were found. Acidobacteria_bin4 encoded several bioactive compounds with antifungal, antibacterial, and antitumor activities, such as stigmatellin, oxazolomycin, carbapenem, myxothiazol, and roseoflavin. These results suggest that the contexts of genes encoding secondary metabolites were distinct in different taxa. Our data provided guidance for targeting the particular biosynthesis clusters of bioactive compounds in the mangrove ecosystem, that is, the importance of considering the preference of different microbial taxonomic information.

**TABLE 2:** Information on three nearly complete *Acidobacteria* genomes

Bin ID^*a*^	Genome size (Mb)	GC content (%)	Completeness (%)	Contamination (%)	No. of BGCs	No. of BGCs in different types
Acidobacteria_bin4	7.01	56.62	94.87	1.71	42	22 NRPS, 8 T1PKS, 3 transAT- PKS-like,^*b*^ etc.
Acidobacteria_bin9	9.03	44.75	90.16	5.3	58	46 NRPS, 8 T1PKS, 2 NRPS-like, etc.
Acidobacteria_bin15	8.20	47.77	91.18	8.12	71	55 NRPS, 8 T1PKS, 3 bacteriocin, etc.

a ID, identifier.

b transAT, *trans*-acyltransferase.

10.1128/mSystems.00851-19.9 TABLE S6 Taxonomic information of 602 newly reconstructed bins. Download Table S6, XLSX file, 0.04 MB. Copyright © 2020 Liao et al. 2020 Liao et al. This content is distributed under the terms of the Creative Commons Attribution 4.0 International license.

**FIG 6:**
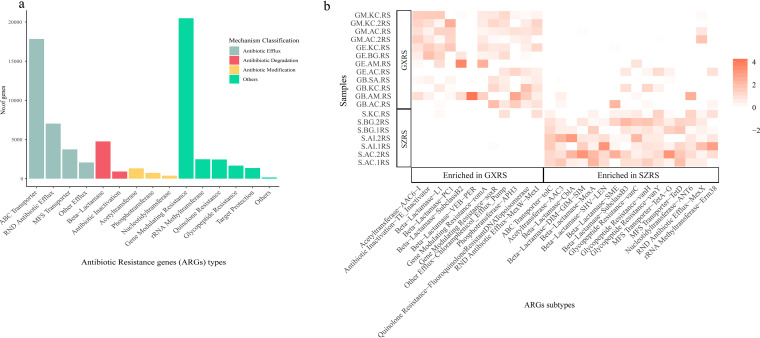
Microbial bioactive secondary metabolites in the mangrove ecosystem. (a) Distribution of biosynthesis gene cluster types. (b) Enrichment of ARG subtypes between SZRS and GXRS.

Based on the observations above, different types of antibiotics can be found in BGC compositions, indicating that the wide distribution of antibiotic resistance genes (ARGs) must be acquired to survive in the mangrove root microbiome. We mined all unique genes with a set of curated hidden Markov models for antibiotic-resistant proteins from the Resfams database (28). A total of 67,278 unique ARGs were found in our data, indicating a diverse set of antibiotic-resistant microbes in a mangrove ecosystem. According to the mechanism classification, the class of gene modulating resistance with a gene number of 20,483 was the most abundant antibiotic-resistant genes, followed by ATP-binding cassette (ABC) transporter (17,832), resistance-nodulation-cell division (RND) antibiotic efflux (7,025), beta-lactamase (4,749), and major facilitator superfamily (MFS) transporter (3,732) (Fig. 6a). Eight types of ARGs associated with rRNA methyltransferase, quinolone resistance, glycopeptide resistance, target protection, acetyltransferase, antibiotic inactivation, phosphotransferase, and nucleotidyltransferase were also present. This finding may highlight the coexistence of antibiotic resistance in mangrove sediment microorganisms. The top six abundant subtypes of ARGs in both locations were *vanR*, *macB*, *vanS*, *msbA*, fluoroquinolone resistance, and RND antibiotic efflux pump. However, the number of AR genes found in the Shenzhen mangrove was 55,412, which was much higher than the number found in Guangxi (39,650). Comparative analysis also showed that 16 subtypes of ARGs were enriched in SZRS, such as *tolC*, AAC3, CblA, DIM-GIM-SIM, MoxA, *vanC*, *vanH*, TetA to TetG, and TetD, which mainly belong to the group of beta-lactamases, glycopeptide resistance, MFS transporter, and other types. Meanwhile, 12 ARG subtypes enriched in GXRS, such as AAC6−I, L1, PC1, VEB-PER, *romA*, and *soxR*, which mainly belong to the group of beta-lactamases, gene modulating resistance, and other types (Fig. 6b, Wilcoxon rank sum test, adjusted *P* value < 0.05). The subtypes of ARGs without any differences between the two sites were mainly comprised of subtypes such as *vanR*, *macB*, *vanS*, *msbA*, RND antibiotic efflux pump, and others, corresponding to gene modulating resistance, ABC transporter, and RND antibiotic efflux groups.

## DISCUSSION

Microbes are important for maintaining ecosystems because they play essential roles in biogeochemical cycles. In the mangrove ecosystem, microbial diversity and functional activity provide the foundation for its high productivity and uniqueness (10). Therefore, it is valuable to create a comprehensive gene catalogue to systematically describe the microbial composition and functionalities in the mangrove forest. The microbial composition of the mangrove ecosystem is strongly influenced by various factors, including vegetation types, biogeographical, and anthropogenic factors (9, 10). Thus, the root-associated microbiomes, including rhizosphere, rhizoplane, and bulk soil from representative mangrove plants in each mangrove ecosystem should be collected to capture most of the gene information of the mangrove microbiome. In this study, we constructed an 87M microbial gene set from rhizoplane, rhizosphere, and bulk soil samples from two mangrove ecosystems located in two different provinces of southern China, providing a fundamental basis to explore the microbial taxonomic composition and functionalities of mangrove forests.

In our study, bacteria and archaea were the most abundant domains recovered from all rhizosphere samples, and very few fungal sequences were acquired. However, a large number of fungal sequences could be detected by fungal internal transcribed spacer (ITS) or 18S rRNA gene amplicon sequencing (29, 30), whereas fewer were captured in the studies using metagenome sequencing (19, 21, 31, 32). One of the possible reasons for the finding discussed above is that the content of bacteria is indeed higher than fungi in the soil. In addition, the complexity of the fungal genome (repeated sequence, heterozygosity, etc.) is much higher than that of bacteria, which makes it more difficult to capture complete fungal sequences during metagenome assembly. For the comparative analysis, the fungal data are usually not sufficiently representative, and therefore, only the bacterial taxonomic and functional composition are analyzed in most metagenomics studies (19, 31, 33, 34).

Recent studies in mangrove microbiomes from different geographical locations and different host plants have conclusively demonstrated that the mangrove microbiome is composed of a few dominant phyla, mainly *Proteobacteria*, *Bacteroidetes*, *Chloroflexi*, *Actinobacteria*, and *Planctomycetes* in bacteria, *Crenarchaeota*, *Thaumarchaeota*, and *Euryarchaeota* in archaea, and Ascomycota in fungi, which is highly consistent with our study (see Table S7 in the supplemental material). Although the dominant phyla in different mangroves were similar, the genus composition and proportion varied across different geographical locations. For example, the top five most abundant genera were *Sulfurovum*, *Nitrospira*, *Desulfobacterium*, *Desulfuromonas*, and *Planctomyces* in Mai Po Ramsar Wetland from Hong Kong, China (6), while *Burkholderia* and *Geobacter* were common in mangrove forests across Kerala, India (35). Moreover, *Sulfurovum*, *Nitrospira*, and *Desulfobulbus* were the most abundant genera in a pristine mangrove of Yunxiao, China. Of these genera, *Syntrophobacter*, *Sulfurovum*, *Nitrospira*, and *Anaerolinea* potentially drove the coupling of carbon, nitrogen, and sulfur cycling (2). These genera were also found in SZRS and GXRS.

10.1128/mSystems.00851-19.10 TABLE S7 Summaries of the taxonomic and functional patterns of mangrove microbiomes in different locations and comparison with the results of our study. Download Table S7, XLSX file, 0.02 MB. Copyright © 2020 Liao et al. 2020 Liao et al. This content is distributed under the terms of the Creative Commons Attribution 4.0 International license.

Metagenomic study and GeoChip-based analysis of mangrove associated microbiome have provided a snapshot of microbial functional composition and geographical distribution in some specific areas (8, 36), but the data are limited. Key functional gene categories in mangrove microbial communities mainly involved carbon fixation, carbon degradation, methane generation, nitrogen fixation, nitrification, denitrification, ammonification, nitrogen reduction, sulfur metabolism, metal resistance, antibiotic resistance, and organic contaminant degradation (1, 8). The biochemical transformation functions of the mangrove microbiome in carbon, nitrogen, and sulfur have mostly been recorded in the Yunxiao mangrove and Brazilian mangroves (1, 2, 10). However, variance in the whole functional composition of mangrove ecosystems remains unclear in various locations, especially the locations impacted by anthropogenic activities. Shenzhen Mangrove Nature Reserve in Guangdong Province was an urban forest, near the estuary, which was considered a convenient site to discharge municipal sewage and industrial wastewater. A large number of bacteria involved in sulfate reduction and archaea involved in methanogenic metabolism were enriched in the Guangxi mangrove. The presence of these specific genera was an indication that methane metabolism and the sulfite cycle were rich in the Guangxi mangrove ecosystem. Meanwhile, mainly AOA enriched in the Shenzhen mangroves might drive the nitrogen cycling. Actually, the enriched functional pathways shifted between Guangxi and Shenzhen mangroves. Obviously, the key enzymes related to nitrogen metabolism were enriched in Shenzhen mangroves, specifically the reactions related to dissimilatory nitrate reduction, assimilatory nitrate reduction, denitrification, and nitrification. However, the key enzymes related to sulfite metabolism and methane production were enriched in Guangxi mangroves. The shifts in microbial functionalities between the mangroves may reflect the change in soil environment due to human activities. The mangrove sediments are often characterized as carbon rich, sulfur rich, and nitrogen limited (2). Aerobic respiration and sulfate reduction are considered to be the major pathways of mangrove-derived carbon degradation (37). An earlier study from mangroves near a wastewater area (38) reported that sulfate respiration functions were mainly attributed to the class of Deltaproteobacteria, represented by *Desulfobulbaceae*, *Desulfobacteraceae*, *Desulfarculaceae*, and so on. But in the mangrove along the coast of Beibu Gulf in Guangxi (7), the sulfate reduction bacteria *Desulfococcus* showed higher abundance at both a pristine site (Yuzhouping) and anthropogenic site (Beilun Estuary Nature Reserve) in comparison with other sites. In the study of the Brazil mangrove microbiome (1), both metagenome and metatranscriptomic data showed that sulfate reduction genes *apr*AB and *dsr*AB were higher in the Mgv04 sample collected from the natural site than in the Mgv03 sample from the city-impacted site, which was in accordance with our result. Collectively, sulfate reductive taxa and the capacity of sulfate reduction in mangrove microbiome differ with locations and with conditions under anthropogenic activities. In our study, SRB were observed in both locations, while the relative abundance of SRB in Guangxi mangrove was relatively higher than in Shenzhen. However, what drives the observed variation cannot be fully explained by our data and experimental setting alone. The abundance of SRB can be strongly affected by reservoir temperature, pH of the brine formation, redox status, and sulfur content of the soil (39, 40). In fact, through an analysis of the distribution of sulfur in several mangrove wetlands in China, the previous study concluded that total sulfur content in Guangxi was relatively higher than in Shenzhen (41). We speculated that the higher sulfur content might be another cause for the enrichment of SRB in Guangxi mangroves, including the low oxygen and high organic matter content.

The differentially enriched pathways were also detected in bulk soil and rhizosphere samples. Most functions of the core rhizosphere have also been found in the citrus and barley root microbiome (19, 20), indicating the adaptive features of root niches. The nutrients of mangrove sediments were influenced by periodic tides of ocean water. The comparison of our data to data sets from the soil and oceanic surface water samples resulted in a higher portion of shared genes with the land plant microbiome, although the mangrove metagenome is highly dissimilar in general (<1% shared genes). However, on the whole, the mangrove metagenome still has its particularity, for example, eight pathways were commonly enriched in a mangrove ecosystem compared with land and sea ecosystems. This finding suggests that the material flow and energy flowing with high productivity, return rate, and decomposition rate in mangrove ecosystems are driven by the microorganisms.

Mangrove microbes contain the potential for producing a large number of secondary metabolites. We predicted 3,622 and 1,288 gene clusters of secondary metabolites from metagenome data and newly reconstructed genomes. However, only 761 and 256 of these predicted BGCs, accounting for 21.01% and 20.08% of the total, could be annotated in the MIBiG database. This finding indicated that there are numerous unknown novel bioactive compounds to be discovered. The most common and abundant classes of BGCs identified in bins were similar to those found in the metagenome data. However, the classes of BGCs found in the genomes varied across the taxonomic group. Moreover, the *Acidobacteria*, *Myxococcota*, *Cyanobacteria*, and *Actinobacteria* possessed higher median values of BGCs per genome than other phyla. These results indicate the differential genomic contexts of mangrove microbes for biosynthesis clusters. When we targeted particular compounds from microbes, it was very important to consider their taxonomic information. Traditional methods for discovery of bioactive compounds have used culturable microbes from which novel bioactive metabolites are detected with relatively low efficiency. The biosynthesis gene cluster prediction from metagenome assembly and reconstructed genomes of mangrove microbiome allowed us to obtain the genetic information of largely unknown secondary metabolites from uncultured microbes. This BGC data set can be of interest in exploring candidate gene clusters for antibiotic and antitumor activity.

In addition, the antibiotic resistance genes reflect the presence of a large number of potentially antibiotic-resistant microorganisms in this environment. The possible causes leading to the presence of antibiotic-resistant microorganisms may be attributed to exposure to the environment with a high diversity of antibiotic compounds or may be a self-protecting mechanism against the secondary metabolites produced. One interpretation focuses on the direct biosynthesis of these antibiotics by the mangrove microbiome, and the other could relate to input from industrial waste and sewage. Specifically, the number of ARGs found in the Shenzhen mangrove was 55,412, which is much higher than the number found in Guangxi (39,650). Moreover, the subtypes of ARGs enriched in Guangxi differed from those of Shenzhen. The differences in ARGs between the mangroves of two locations not only reflect the differences of the antibiotic-resistant microorganisms but also the potential risks of introducing antibiotics into the environment through human activities.

Collectively, the gene catalogue of the mangrove ecosystem was useful for interpreting the taxonomic diversity and functional structure of the mangrove microbiome. The results shed light on the possible role of microbial organisms in mangrove sediments. At the same time, the potential impact of human activities on microbial communities and biochemical cycling in mangrove ecosystems became clear. Our study provides a possibility for monitoring the dynamic changes of the mangrove ecosystem through microbial changes. The data set of unknown secondary metabolism gene clusters was also helpful in guiding the discovery of locational bioactive substances.

## MATERIALS AND METHODS

### Sample collection.

Two well-separated mangrove forests located in Guangdong and Guangxi provinces along the southern coast of China were selected for the present study. Futian Mangrove Nature Reserve in Shenzhen of Guangdong Province is a national nature forest in the hinterland of the city, which is referred to as a mangrove forest impacted by anthropogenic activities. The mangrove wetlands of Beilun Estuary National Nature Reserve and Hepu and Maoweihai Mangrove Nature Reserve in Guangxi Province are located far from any industrial or residential areas and are considered to be pristine forests (7). Site SZ is from the Futian Mangrove Nature Reserve in Shenzhen of Guangdong Province. Site GB is located in Hepu County of Beihai city. Site GE is located in Beilun Estuary National Nature Reserve in Fangchenggang, a coastal city. Site GM is located in Maoweihai Mangrove Nature Reserve in Qinzhou city.

The rhizoplane, rhizosphere, and associated bulk soil samples from four representative mangroves (*Kandelia candel*, *Bruguiera gymnorrhiza*, *Acanthus ilicifolius*, and *Aegiceras corniculatum*) in Shenzhen mangrove were collected. In addition, the rhizosphere and bulk soil samples from five representative mangroves (*Avicennia marina*, *Aegiceras corniculatum*, *Kandelia candel*, *Bruguiera gymnorrhiza*, and *Sonneratia apetala*) in Guangxi mangroves were sampled (see Table S1 in the supplemental material). Soil samples, including rhizosphere and rhizoplane samples were obtained using the same method as described in a previous study (42). The bulk soil samples in our study were samples from an unplanted area in the mangrove forest. All the samples were immediately stored at −20°C and transported back to our laboratory for DNA extraction. In order to enlarge our mangrove microbial unique gene set, a total of 39 samples, including all rhizosphere, rhizoplane, and bulk soil were used to construct the nonredundant gene set. We filtered six rhizoplane samples of Shenzhen mangroves as the limited number and only collected in Shenzhen mangroves. We also trimmed four samples collected from the constructed wetland system in Shenzhen mangroves, which were used to treat wastewater. Only the rhizosphere and bulk soil samples collected in the mangroves were used for subsequent comparative analysis, comprising 12 rhizosphere soil and 8 bulk soil samples from Guangxi mangroves and 7 rhizosphere soil and 7 bulk soil samples from Shenzhen mangroves.

### DNA extraction and sequencing.

At first, the rhizoplane samples were washed off from the root with a mixture of 1× phosphate-buffered saline (PBS) and adsorbent Silwet-77. Then 0.5 g of each sediment sample was used to extract the total DNA by using a PowerSoil DNA isolation kit (Mobio Labs, Inc., Solana Beach, CA, USA) according to the manufacturer’s protocol. The DNA from the rhizosphere soil and bulk soil samples was extracted from 0.5-g sediments using the same method. Last, all DNA samples were sequenced on the BGISEQ500 platform using paired-end sequencing strategy (43). The total amount of sequencing data for all the samples reached 1,701 Gb, with a mean of 43.6G for each sample.

### Read processing and *de novo* assembly.

All the raw data were trimmed by SOAPnuke (v1.5.2) (44) by removing adaptor sequences and trimming and removing low-quality reads at BGI-Shenzhen, China. Twenty-four samples, including 19 samples from Shenzhen mangroves and five samples from Guanxi mangroves, were separated into three groups based on their microbial community similarity calculated by Mash (45). All the metagenomic reads from each group were pooled together to perform *de novo* assembly using Megahit version 1.0.3 (46) with the meta-large preset parameter. The remaining 15 samples from Guangxi mangroves were assembled separately with the same parameters. A total of 0.72M assembled contigs were generated with a total length of 57 Gb. Then all clean reads were mapped against the contigs to calculate the read utilization rate. An average of 26% of the total reads were used to construct the contigs.

### Gene prediction and the nonredundant gene set construction.

Genes were predicted over the contigs with the length of >300 bp by Prodigal (47) using meta mode, resulting in approximately 186M original genes. Due to the lengthy time consumption of computing resources, using nucleotide sequences to construct the nonredundant gene set, we constructed a unique gene set using an amino acid sequence by CD-HIT (48) with the same coverage parameter 0.9 and the identity cutoff of 95% as described in a previously published paper (49). Finally, we had a nonredundant gene set, including 87,794,915 genes with a total length of more than 41.6 Gb.

### Gene profile, functional profile, and taxonomic profile.

Gene functions were annotated by aligning protein sequences of nonredundant genes against KEGG (version.81) (50) using Diamond (51), and the blast results meeting the criterion “hit score > 60, win score = 1” were retained. Taxonomic annotations were implemented by mapping genes to the NCBI Non-Redundant Protein Sequence Database with Diamond (51), and the taxonomic classifications of each gene were determined by filtering the alignments, using an in-house lowest common ancestor (LCA) pipeline. The filtered reads were mapped to the nonredundant gene set by Bowtie2 (52) with default parameters. The absolute abundance of each gene was calculated as described in the previous study (53). The KO profile and taxonomic profile were generated by summing up the absolute abundance of genes that affiliated with the same KO and the same taxonomy, respectively.

### Comparison analyses of different gene sets, taxa, and pathways.

The normalization steps were performed before making taxonomic and functional KO comparison between the Shenzhen and Guangxi samples. The count data of the features (phyla, genera, and KOs) were normalized using DESeq2 package in R software (54). Then the relative abundances of the items in each sample were converted by dividing by the sum of normalized counts. The differential analysis of the microbial genes and taxa between the different habitats was performed using the Wilcoxon rank sum test with an adjusted *P* of <0.05 based on the relative abundance profile.

In order to select the representative taxa in different mangrove ecosystems, we screened the general taxa with an occurrence frequency of more than 80% in the Shenzhen rhizosphere samples or Guangxi rhizosphere samples, respectively. Then the differential genera (Guangxi rhizosphere-enriched genera and Shenzhen rhizosphere-enriched genera) were detected based on a relative abundance table using Wilcoxon rank sum test in R. The *P* values were adjusted by the Benjamini and Hochberg (BH) method.

The KO profile was normalized by using DESeq2 (54), and then the pairwise comparison of 19 rhizosphere samples with corresponding bulk soil samples was analyzed to reveal the functional pathways in mangrove rhizosphere microbes. Seven SZRS samples and 12 GXRS samples were compared to reveal the functional structural differences between the two locations. The differentially enriched KOs were calculated based on the normalized abundance using the Wilcoxon rank sum test with an adjusted *P* of <0.05.

Functional comparisons of 19 mangrove rhizosphere samples with 20 citrus rhizosphere samples and 63 Tara Ocean surface samples (prokaryote-enriched fractions 0.22 to 1.6 mm, 0.22 to 3 mm) were analyzed to find the uniqueness of mangrove rhizosphere microbe. We first selected the coincident KOs in both groups from the KO profile data sets of three different environments. Then the absolute abundance of each KO in the sample was normalized by dividing with the sum of all selected KO abundances. The differentially enriched KOs were calculated based on the normalized abundance using the Wilcoxon rank sum test with an adjusted *P* of <0.05. Then, the differentially enriched KEGG pathways were identified as described in the previous study (55). KEGG pathways with reporter score (z-score) of ≥1.7 could be considered significantly differentiating pathways.

We compared the unique gene set of mangrove root microbiota with citrus, ocean, rice (22), and tomato (23) by CD-HIT (48) with the same coverage parameter 0.9 and the identity cutoff 0.95.

### Genomic binning.

Contigs longer than 2 kb were clustered for genome bins. Bowtie2 (52) was used to cross-map reads from all assemblies to the contigs from assembly to obtain the coverage profiles. Then the coverage profiles were used in MetaBAT2 (56) to run binning on all samples individually. Subsequently, the completeness and contamination of all bins were assessed by CheckM (57). After filtering for completeness of ≥70% and contamination of ≤10% or completeness of ≥50% and contamination of ≤5%, 656 draft genomes were left. To obtain nonredundant bins, dereplicating was performed using dRep (58) with options: dRep dereplicate outdir2 -g –S_algorithm gANI -nc 0.6 –noQualityFiltering. A final set of 602 nonredundant bins was obtained. The taxonomic assignment of these bins was generated using GTDB-Tk v0.2.2 (59) with the option of gtdbtk classify_wf. Then we mapped the clean reads of each sample to the 602 bins using Bowtie2 (52).

### Bacterial biosynthesis gene cluster analysis.

In response to the need for searching for a more robust predictor of chemical contents, we predicted the secondary metabolite BGCs by using antiSMASH5.0 (60) with the assembly contigs larger than 10 kb from the mangrove metagenomes and 602 bins. We used the local version of anti-SMASH5.0 to predict gene cluster sequences with the following parameters: --genefinding-tool prodigal --cb-general --cb-knownclusters --cb-subclusters --asf --pfam2go --smcog-trees. In order to find out what the products are exactly, the predicted biosynthetic gene clusters were aligned with those reported in the MIBiG repository (61) using BLASTP. The closest homolog cluster was selected based on the highest cumulative BLAST score.

### Antibiotic resistance gene search.

ARGs were identified through the Resfams database by HMMER (28). Then we filtered the hits according to the E value less than 1e−5 of the full sequence blasting out. For the different hits of the same genes, we selected the best hit with the highest score. ARG profiling was generated by summing up the absolute abundances of genes that affiliated with the same subtypes or types according to mechanism classification. Then we converted the absolute abundance into relative abundance in the sample, and the ARG differential comparisons between 12 Guangxi rhizosphere and seven Shenzhen rhizosphere samples were executed by using the Wilcoxon rank sum test (adjusted *P* value < 0.05), which was the same as the calculation of KOs.

### Statistical analysis.

The count data of the various features (phyla, genera, and KOs) were normalized with the function “estimateSizeFactors” in the package DESeq2 in R (54). Then the differential analysis of the microbial genes and taxa between the different habitats was performed using the Wilcoxon rank sum test with an adjusted *P* of <0.05. The *P* value was adjusted by the Benjamini and Hochberg (BH) method. PCoA utilizing the Bray-Curtis dissimilarity was calculated using the “pcoa’” function in the Ape package. Alpha (α)-diversity measurements were calculated with the “diversity” function, using the “Shannon” method from the Vegan package. Permutational multivariate analysis of variance (PERMANOVA) was performed using the “adonis” function from the Vegan package in R software. Figures such as barplot, heatmap, boxplot, and scatterplot were drawn by R package ggplot2.

### Data availability.

The paired-end (PE) read fastq files of 39 samples have been deposited in the CNSA (https://db.cngb.org/cnsa/) of CNGBdb with accession code CNP0000516 and in NCBI with the accession number PRJNA629394.
