# Supplementary figures and images for: Deciphering the Microbial Taxonomy and Functionality of Two Diverse Mangrove Ecosystems and Their Potential Abilities To Produce Bioactive Compounds
Source: mSystems. 2020 Oct 27;5(5):e00851-19. doi: 10.1128/mSystems.00851-19 (PMC7593590; doi:10.1128/mSystems.00851-19)

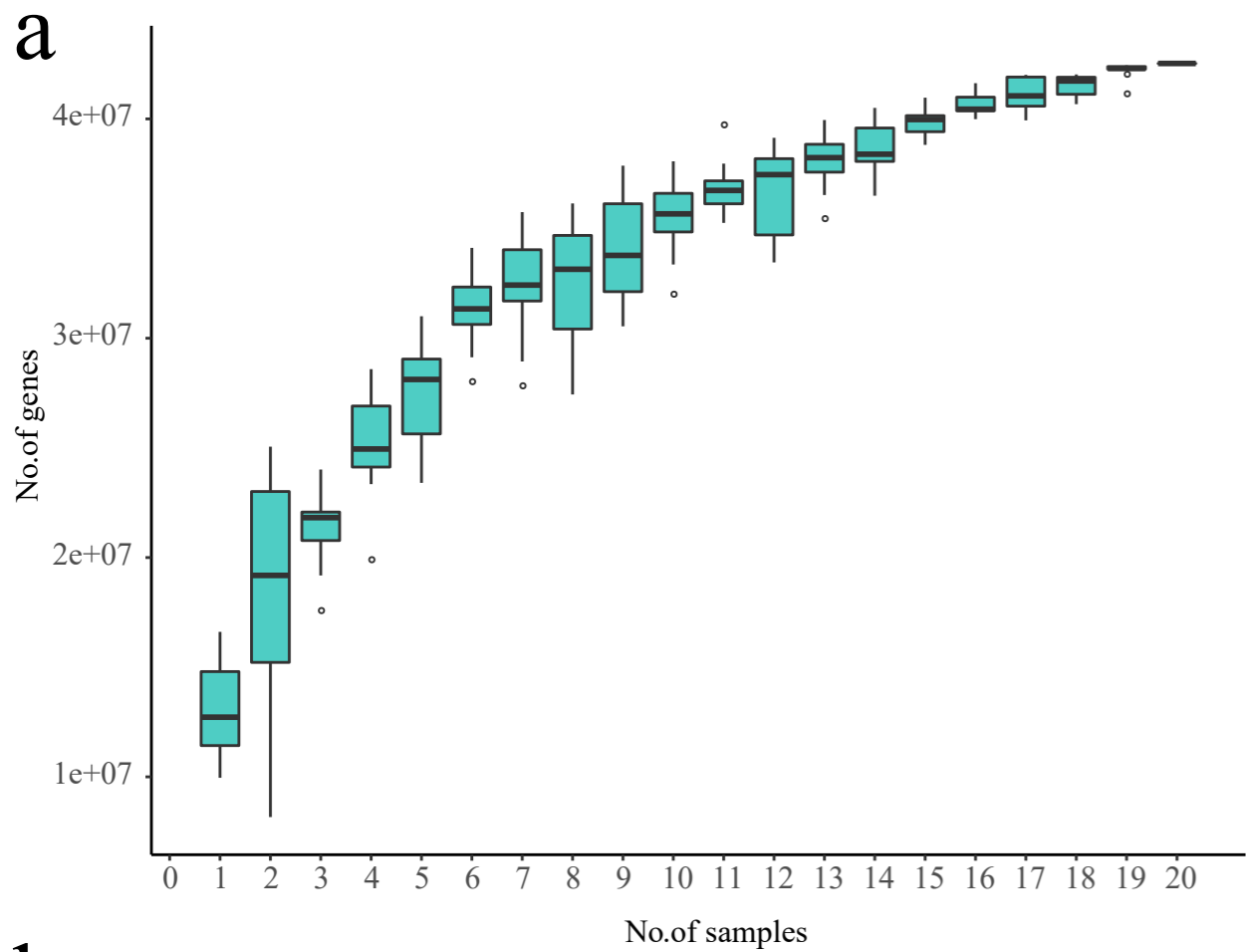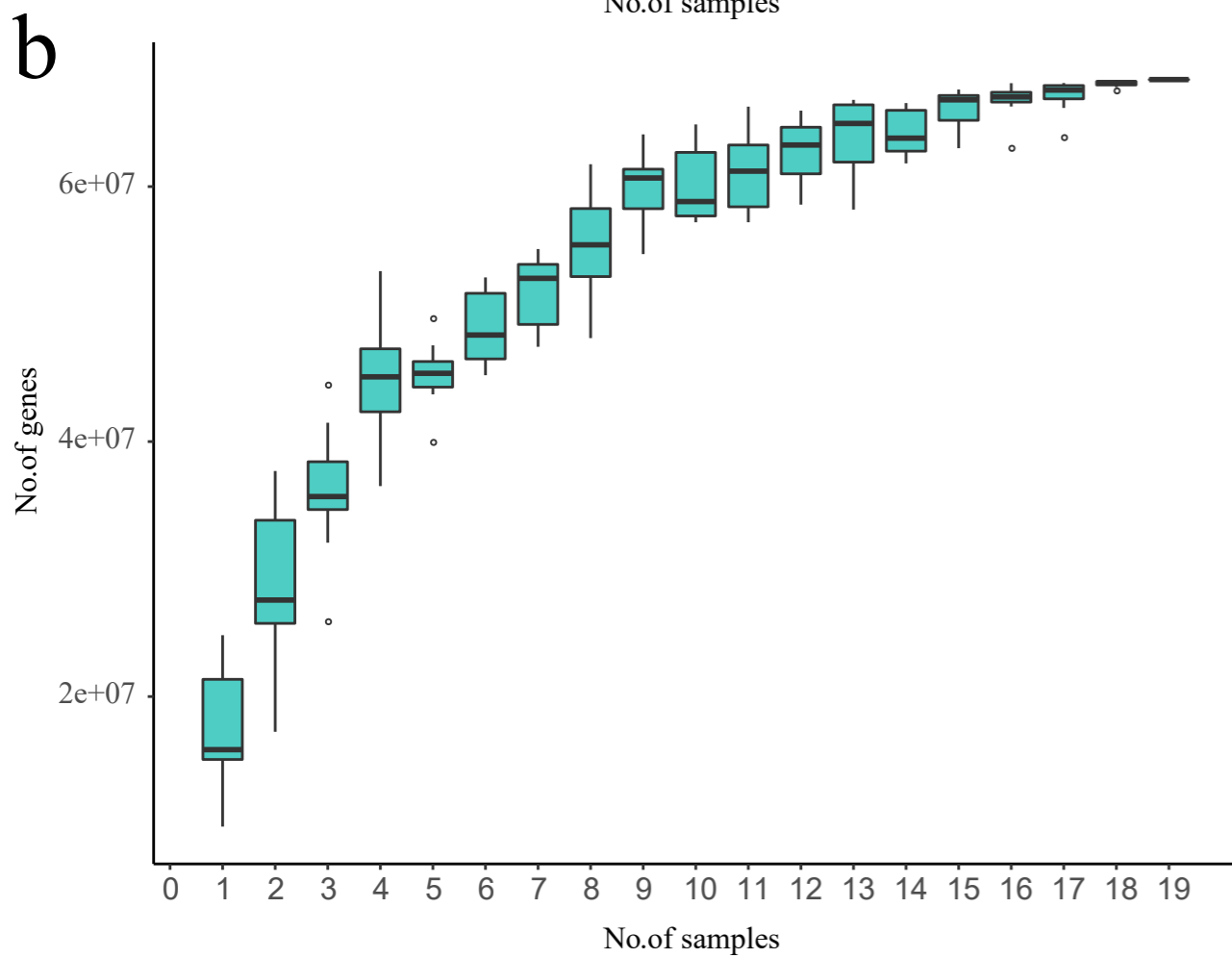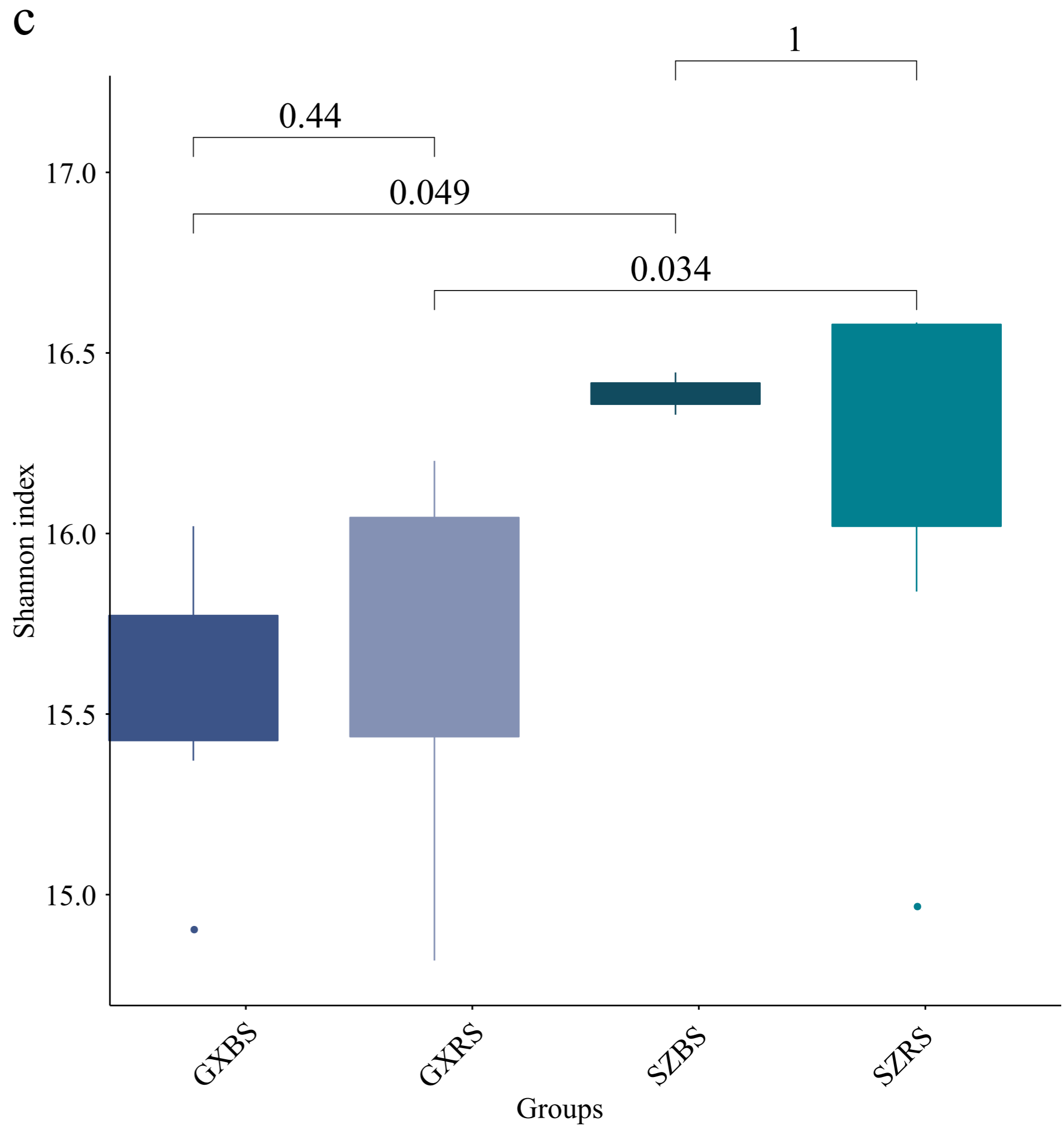

Supplement: FIG S1 [file mSystems.00851-19-sf001.pdf]

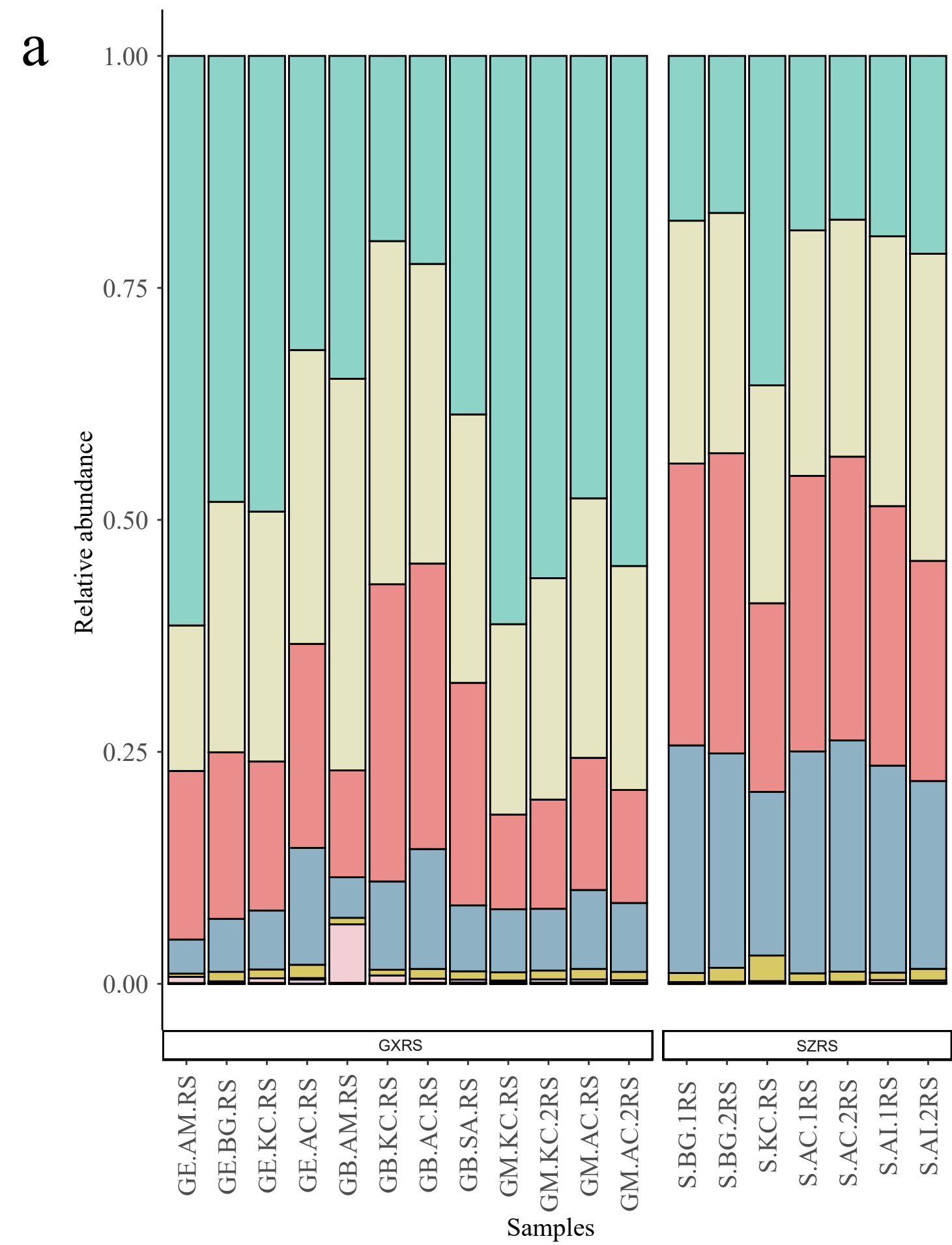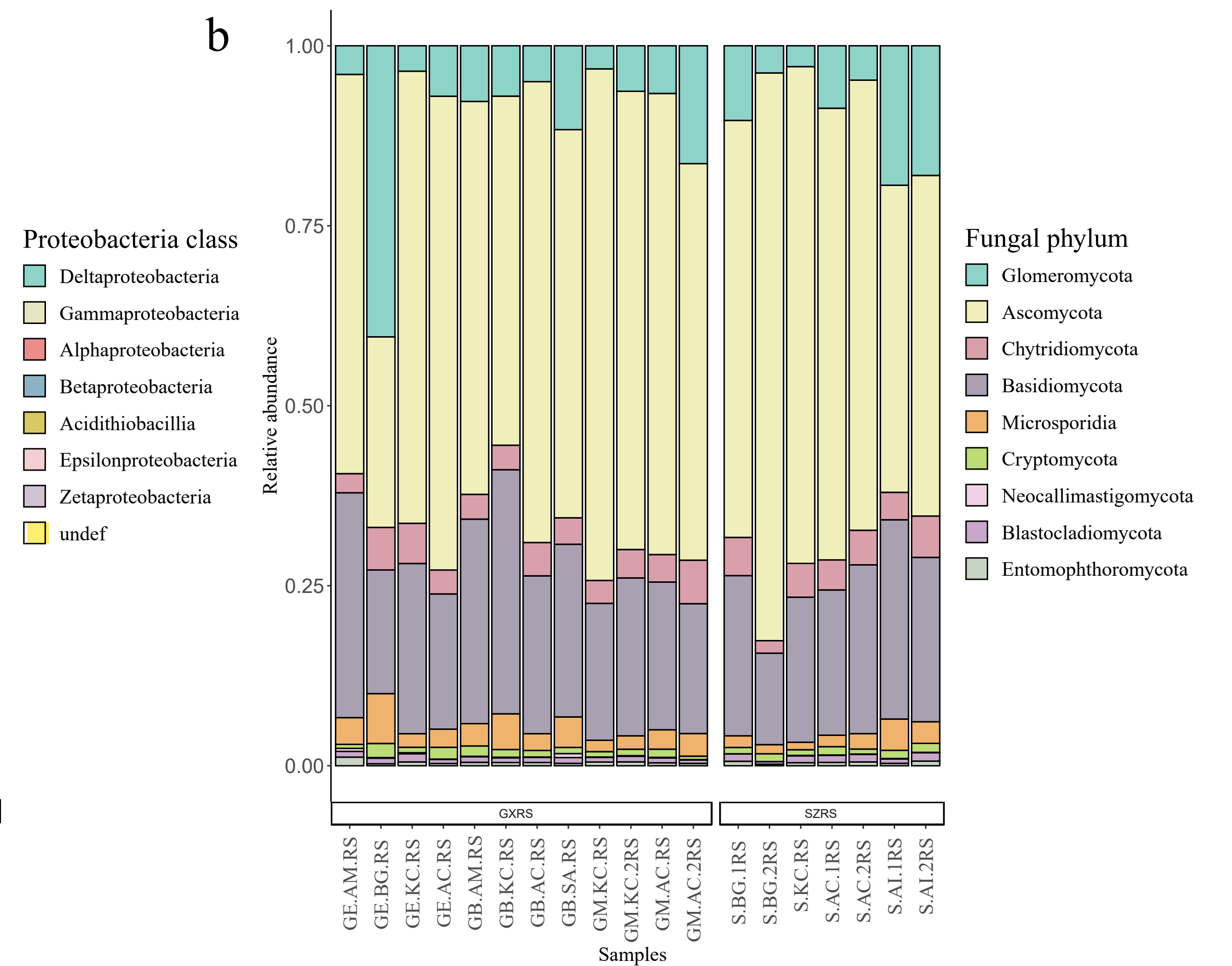

Supplement: FIG S2 [file mSystems.00851-19-sf002.pdf]

a Methane metabolism ko00680

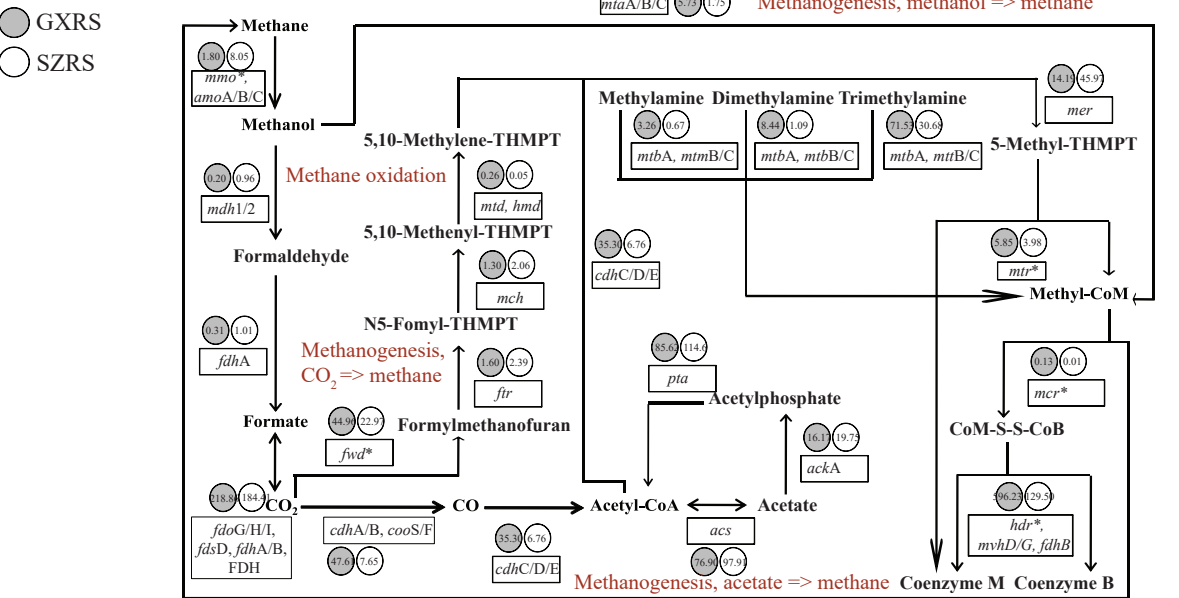

b Nitrogen metabolism ko00910

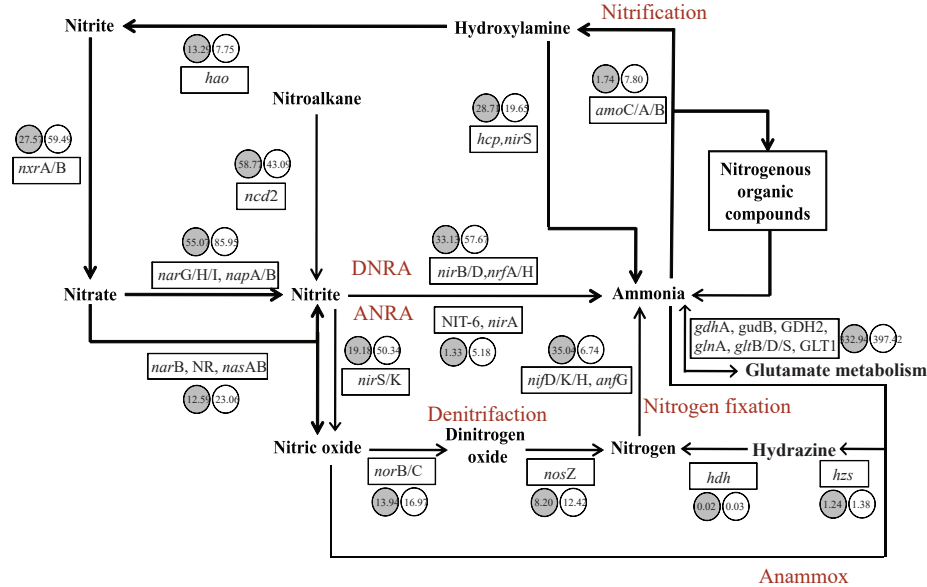

c Sulfur metabolism ko00920

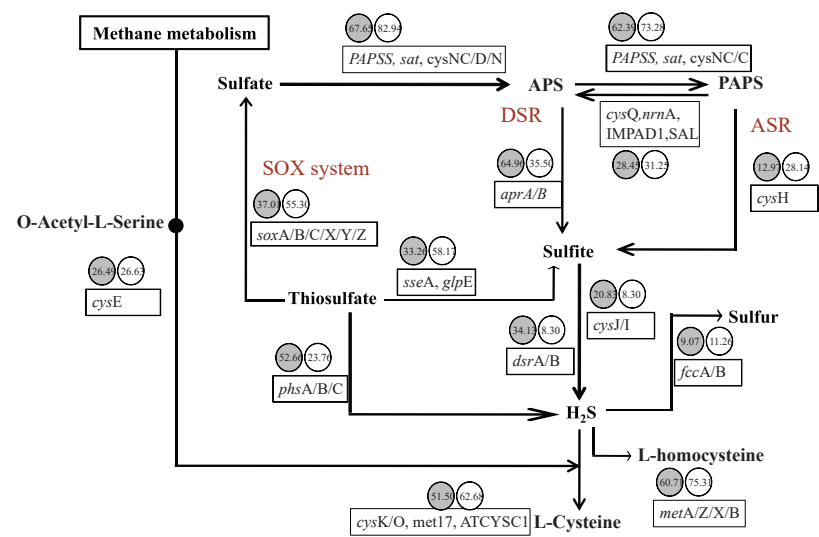

Supplement: FIG S3 [file mSystems.00851-19-sf003.pdf]
